# Acetylated microtubules are required for fusion of autophagosomes with lysosomes

**DOI:** 10.1186/1471-2121-11-89

**Published:** 2010-11-22

**Authors:** Rui Xie, Susan Nguyen, Wallace L McKeehan, Leyuan Liu

**Affiliations:** 1 Center for Cancer and Stem Cell Biology, Institute of Biosciences and Technology, Texas A&M Health Science Center, Houston, Texas 77030-3303, USA

## Abstract

**Background:**

Autophagy is a dynamic process during which isolation membranes package substrates to form autophagosomes that are fused with lysosomes to form autolysosomes for degradation. Although it is agreed that the LC3II-associated mature autophagosomes move along microtubular tracks, it is still in dispute if the conversion of LC3I to LC3II before autophagosomes are fully mature and subsequent fusion of mature autophagosomes with lysosomes require microtubules.

**Results:**

We use biochemical markers of autophagy and a collection of microtubule interfering reagents to test the question. Results show that interruption of microtubules with either microtubule stabilizing paclitaxel or destabilizing nocodazole similarly impairs the conversion of LC3I to LC3II, but does not block the degradation of LC3II-associated autophagosomes. Acetylation of microtubules renders them resistant to nocodazole treatment. Treatment with vinblastine that causes depolymerization of both non-acetylated and acetylated microtubules results in impairment of both LC3I-LC3II conversion and LC3II-associated autophagosome fusion with lysosomes.

**Conclusions:**

Acetylated microtubules are required for fusion of autophagosomes with lysosomes to form autolysosomes.

## Background

Autophagy is the major catabolic pathway for degradation of dysfunctional organelles and macromolecules. First characterized in yeast genetically conserved ATG proteins emerged that participate in and regulate the process of autophagy. ATG proteins are grouped into 1) a Class III phosphatidylinositol-3-kinase (PI3K) complex functioning in vesicle nucleation, 2) a serine-threonine kinase complex involved in induction of autophagy, and 3) ubiquitin-like protein conjugating systems ATG12 and ATG8 that promote maturation of vesicles [1].

The mammalian homologue of ATG8 is LC3, an interactive partner of microtubule-associated protein MAP1A/MAP1B [2,3] and C19ORF5 (Xie R, Nguyen S, McKeehan K, Wang F, McKeehan WL, Liu L: Microtubule-associated Protein C19ORF5 Bridges Autophagic Components with Microtubules and Effects Autophagosomal Biogenesis and Degradation, *Submitted*).. The LC3 precursor is truncated to LC3I then conjugated with phosphatidylethanolamine to membrane-associated LC3II mediated by the ATG5-ATG12 conjugate [4,5]. The LC3II-associated isolation membranes mature and fuse with lysosomes to form autolysosomes in which LC3II is degraded along with the cargo of the autophagosome [6]. The autophagic process can be divided into autophagosomal biogenesis and autophagosomal degradation based on the fate of LC3 isoforms [7]. Both LC3I and LC3II are used as markers for autophagy at different steps and levels reveal a balance of biogenesis and conversion/degradation, respectively. Caution is required to interpret the results from immunoblot since the LC3 levels are dynamically altered [8]. Increasing levels of LC3I suggest increased production of LC3I and reduced conversion to LC3II while increasing levels of LC3II indicate enhanced conversion of LC3I to LC3II and impaired degradation through lysosomes. For example, the accumulation of LC3II in cells cultured in Hanks' media has been interpreted as a consequence of autophagic activation based on the assumption that the capacity of lysosomal degradation remains constant [3]. However, such accumulation could also be caused by an impairment of lysosomal degradation. In order to correctly interpret the LC3 immunoblot data, lysosomal inhibitor NH_4_Cl or bafilomycin A1 are used to block autophagosomal degradation in lysosomes to show the total amount of converted LC3II during blockade [9-11]. An increase in the total amount of LC3II in the presence of lysosomal inhibitor indicates an increase of autophagic influx, e.g. more LC3I production and faster conversion to LC3II [12].

Microtubules are polymers of tubulin dimers whose dynamics are regulated by microtubule-associated proteins. They constantly polymerize and depolymerize to facilitate trafficking of organelles along microtubular tracks and chromosomal segregation in mitosis [13-17]. After assembly, microtubules are constantly modified in different patterns to enhance their functions. One type of modification is acetylation that results in acetylated microtubules that recruit molecular motors enabling increased flux of vesicles along microtubular tracks [18,19]. The mammalian autophagic marker LC3 suggests a potential role of microtubules at multiple stages in autophagy. The microtubule-associated proteinsMAP1A/B and C19ORF5 interact with both LC3I and LC3II and facilitate their association with microtubules, suggesting an involvement of microtubules in both autophagosomal biogenesis and degradation [20,21] (Xie R, Nguyen S, McKeehan K, Wang F, McKeehan WL, Liu L: Microtubule-associated Protein C19ORF5 Bridges Autophagic Components with Microtubules and Effects Autophagosomal Biogenesis and Degradation, *Submitted*). Previous reports suggested that microtubules are required for the trafficking of mature autophagosomes [11,22,23]. It is still in debate whether microtubules play a role in autophagosomal biogenesis and subsequent fusion of autophagosomes with lysosomes depends on microtubules [11,22,23].

To decipher roles and types of microtubules in each step of autophagy, we applied a set of microtubule interfering reagents and inhibitors of lysosomal activity to native HeLa cells or HeLa cells stably expressing the autophagic marker GFP-LC3. Using both biochemical and cell biological approaches, we found that regular non-acetylated microtubules are involved in autophagosomal biogenesis but not required for autophagosomal degradation. It is the acetylated microtubules that are required for the fusion of autophagosomes with lysosomes to form autolysosomes.

## Results

### Both stabilization and destabilization of microtubules impairs autophagosomal biogenesis only in mitotic cells

**Figure 1:**
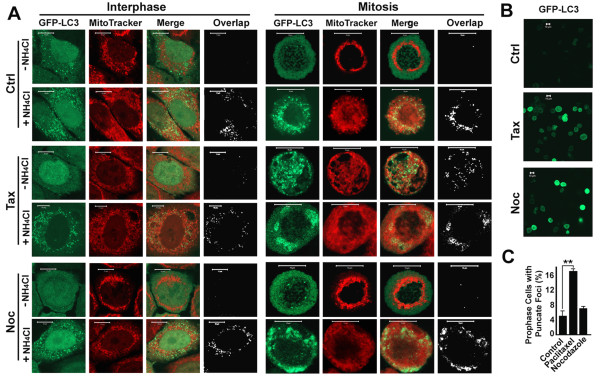
**Only paclitaxel causes accumulation of punctate foci of GFP-LC3 in mitotic cells**. (**A**) Accumulation of GFP-LC3 punctate foci in cells treated with either paclitaxel or nocodazole overnight. Mitochondria are shown in red after exposure to MitoTracker Red CMXRos dye [32,39]. The punctate foci of bright green GFP-LC3 in stably transfected HeLa cells overlapping with weak red punctate foci of dysfunctional mitochondria are indicative of mitophagosomes or mitolysosomes (mitochondria-containing autolysosomes). Colocalized punctate foci in white were revealed by analysis with an ImageJ ColocalizeRGB Plugin. The GFP-LC3 punctate foci overlapping with the much weaker signal of MitoTracker were not detected in the colocalization analysis. Lysosomal inhibitor NH_4_Cl (20 mM NH4Cl) was added simultaneously to preserve the resulting mitophagosomes. Scale bar, 10 μm in all panels. Ctrl, Control, Noc, 10 μM nocodazole; Tax, 10 μM paclitaxel. (**B**) Fluorescent intensity of GFP-LC3 in mitotic cells treated with paclitaxel and nocodazole overnight. (**C**) The percentage of pro-metaphase cells with punctate foci of GFP-LC3 as shown for the mitotic cells treated with paclitaxel overnight in the absence of NH_4_Cl (A). Number of cells with GFP-LC3 punctate foci was counted directly and expressed as a percentage of total mitotic cells. Because of the difference in frequency of mitosis, about 100 mitotic cells in 20 to 50 different fields were scored in the presence or absence of nocodazole or paclitaxel, respectively. The data represent the means and standard errors of three independent experiments. **, p < 0.01 as calculated by Student's T-test.

To investigate impact of microtubules on autophagy, we created a HeLa cell line stably expressing GFP-LC3 that mimics native HeLa cell line in autophagic response [9]. As we previously reported [9], fewer GFP-LC3 punctate foci appeared in premetaphase cells than in interphase cells (Figure 1A, Ctrl). When lysosomal activity was inhibited with NH_4_Cl, both interphase and mitotic cells dramatically increased numbers of punctate foci of GFP-LC3 that largely colocalized with MitoTracker-labeled mitochondria (Figure 1A, Ctrl). Treatment with either paclitaxel or nocodazole blocked the cells in premetaphase that carry high intensity of GFP-LC3 signals (Figure 1B). Examination of individual cells under high-power microscopy revealed that more than 16% of paclitaxel-treated mitotic cells contained GFP-LC3 punctate foci that were colocalized with mitochondria (Figure 1A and 1C). This suggests that paclitaxel but not nocodazole caused accumulation of GFP-LC3 punctate foci and the accumulation only occurred in mitotic cells.

**Figure 2:**
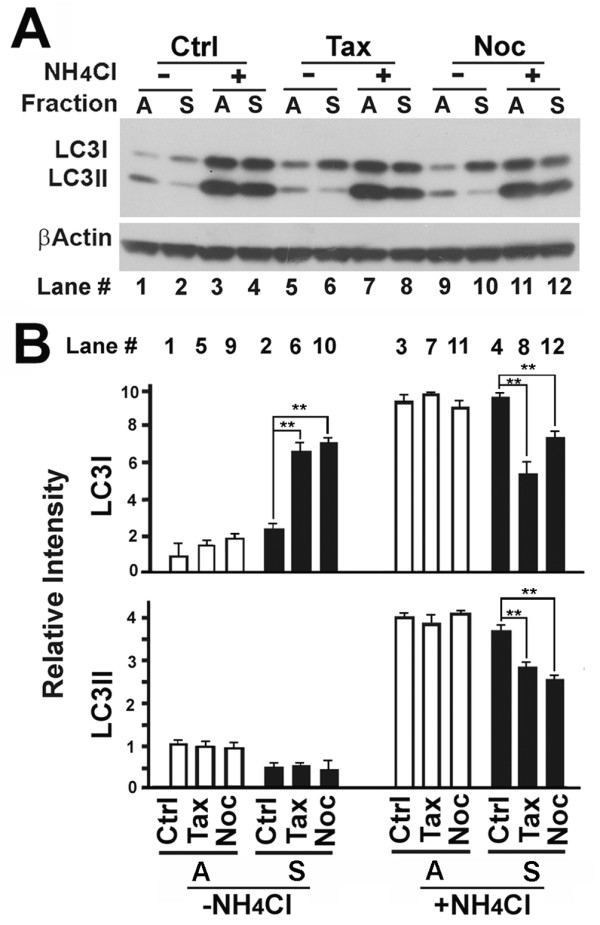
**Interrupting the dynamics of regular microtubules with either nocodazole or paclitaxel impairs conversion of LC3I to LC3II only in mitotic cells**. (**A**) Immunoblot of lysates of fractioned HeLa cells. Mitotic cells were enriched by synchronization with 2 mM thymidine for 20 hr followed by release from the thymidine blockade for 20 hr in the absence or presence of 10 μM paclitaxel or nocodazole as described previously [40]. NH4Cl (20 mM) was added simultaneously with microtubule interfering agents. Loosely attached mitotic cells (S) were harvested by vigorous shake off from the remaining mixture (A) of interphase cells and mitotic cells attached to the dish. (**B**) The relative intensities of LC3I and LC3II bands were calculated from the scanned intensities of bands of three independent experiments as exemplified by immunoblot shown in (A). **, p < 0.01 as calculated by Student's T-test.

The GFP-LC3 pattern described above suggests that nocodazole increased LC3I levels while paclitaxel increased LC3II levels since the punctate foci are usually considered as the LC3II form condensed on autophagosomal membranes. To confirm the idea, we separated the fraction enriched in mitotic cells (S) by shakeoff from the attached fraction that contains both interphase and mitotic cells (A). Immunoblot analysis revealed that mitotic cells contained lower levels of LC3II than interphase cells (Figure 2A, lane 2 vs 1) consistent with previous reports [24,25]. A blockade of lysosomal degradation with NH_4_Cl resulted in increased levels of both LC3I and LC3II to levels that were similar between mitotic and interphase cells (Figure 2A, lane 3,4 vs 1,2). From this we concluded in a previous report that basal levels of autophagy and mitophagy are robust in both interphase and mitotic cells and most autophagosomes that form during the entire cell cycle are efficiently degraded through the lysosomal pathway [9].

Treatment with nocodazole and paclitaxel caused different responses between interphase and mitotic cells. Treatment with either paclitaxel or nocodazole in interphase cells caused a slight increase in LC3I levels and no change in LC3II levels in the absence of NH_4_Cl. However, there was no change in both LC3I and LC3II levels in the presence of NH_4_Cl (Figure 2A and 2B, white bars). The treatments resulted in a 3-fold increase of LC3I levels, but no change of LC3II levels in mitotic cells (Figure 2A, lane 2 vs 6,10, and B, black bars). Accumulation of LC3I suggested either an increased synthesis of LC3I through mechanisms related to transcription, post-transcription or translation of LC3 precursor and conversion to LC3I or reduced conversion of LC3I to LC3II. The levels of LC3I in interphase cells were slightly increased (Figure 2A, lane 1 vs 5,9 and B, white bars) while the levels of LC3I in mitosis were dramatically enhanced upon paclitaxel or nocodazole treatment (Figure 2A, lane 2 vs 6,10 and B, black bars). Although we cannot completely exclude the possibilities that more LC3 mRNA molecules were transcribed or more LC3-I proteins were translated and processed, we believed that such possibilities were unlikely since both mRNA transcription and protein translation are generally suppressed in mitosis. The reduction in total LC3II in either the paclitaxel or the nocodazole-arrested mitotic cells relative to control mitotic cells in the presence of NH_4_Cl (Figure 2A, lane 4 vs 8,12 and B, black bars) further suggested a reduction in net conversion of LC3I to LC3II. Even though punctate foci appeared in more than 16% of paclitaxel-treated mitotic cells (Figure 1C), no difference in LC3II levels was evident. Thus, the paclitaxel-induced GFP-LC3 punctate foci are likely made up of aggregates of primarily LC3I. Others using ATG5-deficient cells have also suggested that that punctate foci containing LC3 do not always represent mature autophagic structures [8]. The ATG5 gene controls the conversion of LC3I to LC3II [26] and its deletion causes accumulation of LC3I. Thus localized accumulation of LC3I on mitochondrial aggregates appears as punctate foci that are less than mature autophagosomes. We suggest that the generation of those punctate foci reflect autophagic failure at the initiation stage rather than autophagy-independent aggregation (Xie R, Nguyen S, McKeehan K, Wang F, McKeehan WL, Liu L: Microtubule-associated Protein C19ORF5 Bridges Autophagic Components with Microtubules and Effects Autophagosomal Biogenesis and Degradation, *Submitted*). In summary, although both paclitaxel and nocodazole impaired the conversion of LC3I to LC3II resulting in accumulation of LC3I, only in mitotic cells did paclitaxel cause the accumulation of LC3I in aggregates.

### Paclitaxel, vinblastine and nocodazole differentially impact microtubular acetylation and structure

Our results above suggest that microtubules affected by paclitaxel or nocodazole support autophagosomal biogenesis but not targeting and fusion with lysosomes. This is consistent with the report by Fass et al. [11]. However, employment of two microtubule-destabilizers nocodazole and vinblastine suggest that microtubules facilitate both autophagosomal biogenesis and fusion of autophagosomes with lysosomes [22]. We examined whether the two drugs interfere with microtubular dynamics differently that might explain the discrepancy.

**Figure 3:**
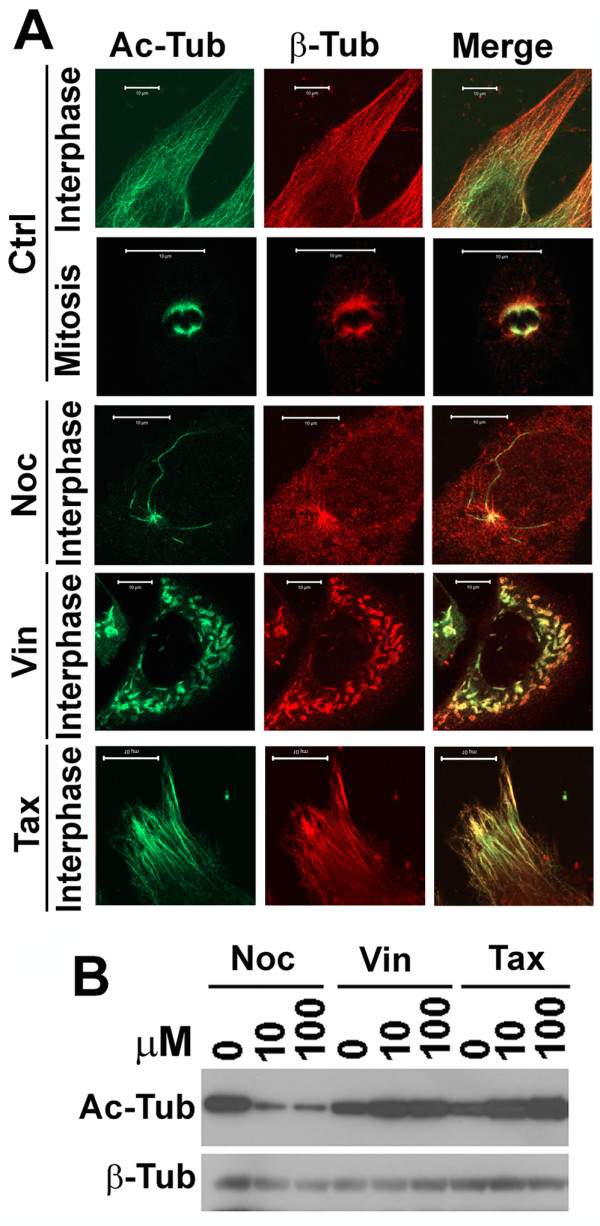
**Impact of paclitaxel, vinblastine and nocodazole on microtubular acetylation and structure**. (**A**) Immunostaining of microtubules in HeLa cells untreated or treated with 10 μM of nocodazole, vinblastine or paclitaxel overnight with antibodies against acetylated α-tubulin and normal β-tubulin. Ac-Tub, acetylated α-tubulin. β-Tub, β-Tubulin. Vin, vinblastine. (**B**) Immunoblotting analysis of acetylated α-tubulin levels in lysates of HeLa cells treated with different concentrations of nocodazole, vinblastine and paclitaxel for overnight.

Acetylated microtubules play an important role in the anterograde trafficking of vesicles [19]. The impact of the tubulin-specific histone deacetylase HDAC6 on the distribution of lysosomes suggested that microtubular acetylation may be important in autophagosome-lysosome fusion [27]. When HeLa cells were stained with a monoclonal antibody against acetylated α-tubulin that is assembled into acetylated microtubules and a polyclonal antibody against β-tubulin that builds up regular microtubules, two sets of microtubular filaments coexisted with the acetylated microtubules that concentrated in the perinuclear region of interphase cells and on the spindles of mitotic cells (Figure 3A). When HeLa cells were treated with increasing concentrations of different drugs, the levels of acetylated α-tubulin were dramatically reduced in the presence of nocodazole, but significantly increased in the presence of vinblastine or paclitaxel (Figure 3B). Examination of the structure of β-tubulin-labeled regular microtubules revealed that both nocodazole and vinblastine caused the depolymerization of regular microtubular filaments. The difference was that microtubules were depolymerized into a diffused state in the presence of nocodazole and short bar-like structures in the presence of vinblastine (Figure 3A, red). In contrast to microtubular depolymerization caused by nocodazole or vinblastine, paclitaxel stabilized microtubules as expected (Figure 3A).

The structures containing acetylated microtubules were affected differently by the drugs (Figure 3A). Regular microtubules were depolymerised, but some fibrilar structures of acetylated microtubules remained (Figure 3A, green) although levels of acetylated tubulin were reduced in the presence of nocodazole (Figure 3B) [28]. Vinblastine caused the depolymerization of not only regular microtubules, but also acetylated microtubules (Figure 3A). Therefore, acetylated microtubules were nocodazole-resistant but vinblastine-sensitive.

### Depolymerization of acetylated microtubules causes accumulation of punctate foci containing GFP-LC3

**Figure 4:**
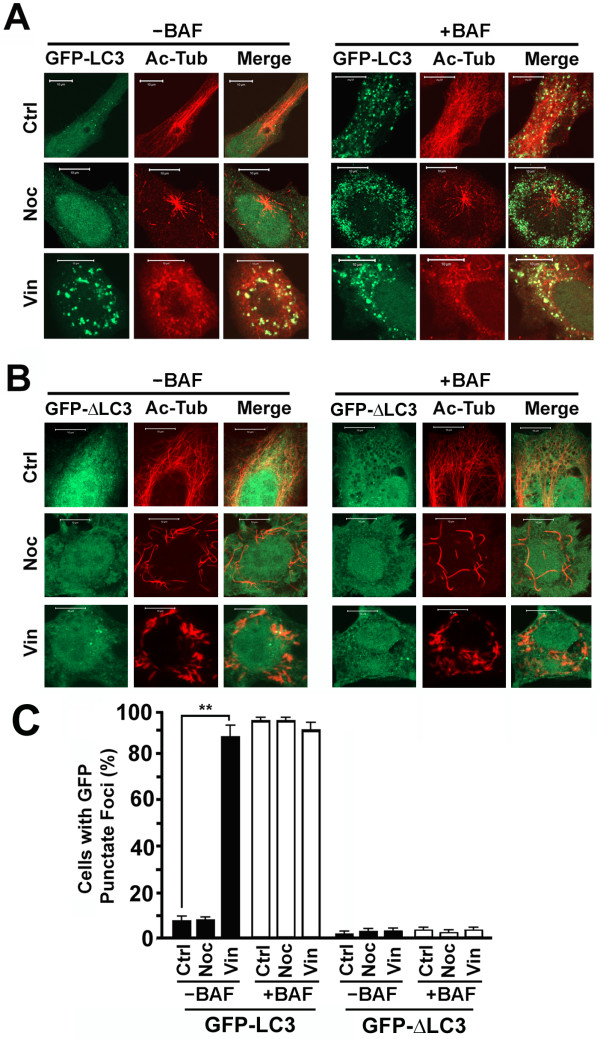
**Vinblastine-induced depolymerization of acetylated microtubules is coincident with accumulation of GFP-LC3 punctate foci**. (**A, B**) Accumulation of GFP punctate foci in HeLa cells stably expressing GFP-LC3 (**A**) or GFP-ΔLC3 (**B**) untreated or treated with either nocodazole or vinblastine in the absence or presence of lysosomal inhibitor bafilomycin A1 (BAF) overnight. The acetylated microtubules are counterstained with anti-acetylated α-tubulin antibody. (**C**) Percentage of GFP expressing cells with more than 10 GFP punctate foci. Only GFP punctate foci occupying an area with diameter greater than four pixels (~0.15 μm) were counted. The data is the average and standard deviation of three independent experiments. **, p < 0.01 as calculated by Student's T-test.

Although both vinblastine and paclitaxel increased levels of acetylated α-tubulin (Figure 3B), vinblastine, but not paclitaxel caused depolymerization of acetylated microtubules. Coincident with the breakdown of acetylated microtubules by vinblastine, the majority of vinblastine-treated cells accumulated GFP-LC3 punctate foci that were colocalized with the dot-like signals of acetylated tubulin paracrystals (Figure 4A). Under the same condition, no significant more GFP punctate foci were formed upon the treatment in the autophagy-defective cell line expressing GFP-ΔLC3 (Figure 4B). Less than 10% untreated or nocodazole-treated GFP-LC3 expressing cells and less than 5% untreated or either nocodazole or vinblastine-treated GFP-ΔLC3 expressing cells accumulated GFP punctate foci (Figure 4C). When lysosomal activity was blocked with another lysosomal inhibitor bafilomycin A1, a large number of GFP-LC3-containing punctate foci accumulated in the untreated or nocodazole-treated cells (Figure 4A) as expected due to the robust basal levels of autophagy [9] while only a few cells expressing the mutant GFP-ΔLC3 accumulate GFP punctate foci (Figure 4B,C). These punctate foci represent true autolysosomes formed through the autophagic machinery that are normally degraded by enzymes in lysosomes in the absence of lysosomal inhibitor. The dramatic difference in the intensities of acetylated microtubules between the untreated and nocodazole-treated cells did not change the number of cells carrying GFP-LC3 punctate foci (Figure 4C). This suggested that a minimal number of intact acetylated microtubules are sufficient to meet demands of trafficking of autophagosomes and lysosomes in order to achieve fusion.

### Vinblastine-induced accumulation of GFP-LC3 punctate foci suggests a blockade of disposal of autophagosomes

**Figure 5:**
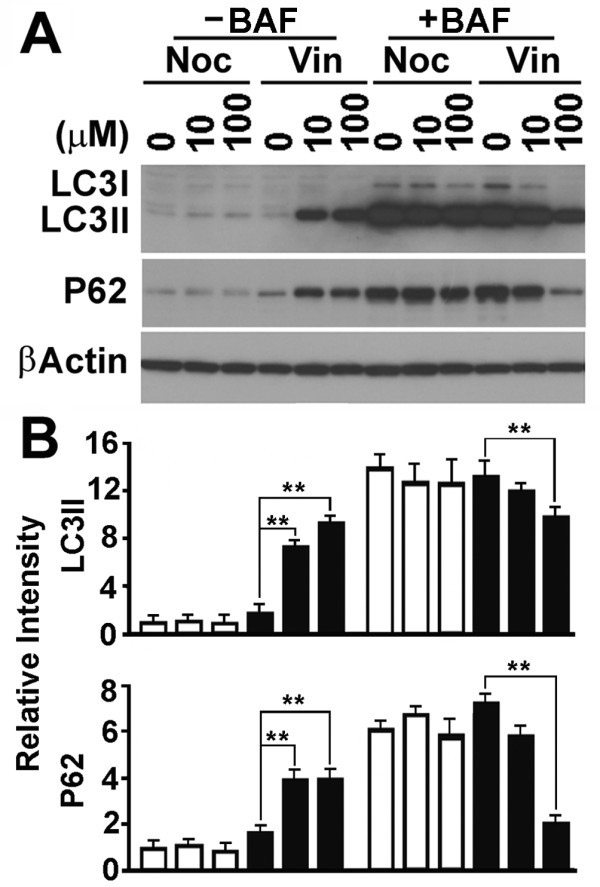
**Only vinblastine causes a blockade of LC3II and P62 degradation**. (**A**) A representative immunoblot of lysates of HeLa cells treated with different concentration of drugs in the absence or presence of lysosomal inhibitor bafilomycin A1 overnight. P62, P62 (SQSTM1). (**B**) Plots of the relative intensity of LC3II and P62 bands detected from three independent blots as represented by the one shown in (A). **, p < 0.01 as calculated by Student's T-test.

The vinblastine-induced accumulation of GFP-LC3 punctate foci may be caused by an activation of autophagosomal biogenesis, a blockade of autophagosomal degradation, or a blockade of conversion of LC3I to LC3II and accompanying localized aggregation of LC3I as indicated by the paclitaxel-induced accumulation of GFP-LC3 punctate foci in mitotic cells (Figure 1 and 2). To distinguish these possibilities, lysates from cells exposed to the different drugs were analyzed by immunoblot. Consistent with the accumulation of GFP-LC3 punctate foci, vinblastine treatment in the absence of lysosomal inhibitor caused a dramatic increase in levels of LC3II and P62, another autophagic marker directly being involved in selective autophagic degradation of ubiquitinated protein aggregates [29] (Figure 5A and 5B, black bars, -BAF). This suggested either an activation of autophagic biogenesis or an inhibition of autophagosomal degradation. Less LC3II and P62 accumulation in the vinblastine-treated cells in the presence of bafilomycin A1 (Figure 5A and 5B, black bars +BAF) confirmed an inhibition of autophagosomal degradation. The cells treated with 100 μM of vinblastine contained similar levels of LC3II, but application of bafilomycin A1 cut P62 in half (Figure 5). These results suggest that autophagosome degradation has been completely inhibited with the high concentration of vinblastine. The reduction in P62 may reflect alternative pathways such as the ubiquitination-proteasome pathway that remains active when autophagy is blocked. In addition, since vinblastine depolymerized both acetylated and regular microtubules (Figure 3A), the efficiency of conversion of LC3I to LC3II was simultaneously reduced in its presence so that the total amount of LC3II generated during the blockade was reduced (Figure 5A and 5B, black bars +BAF).

### The vinblastine-induced blockade of autophagosomal degradation occurs just prior to autophagosome-lysosome fusion

**Figure 6:**
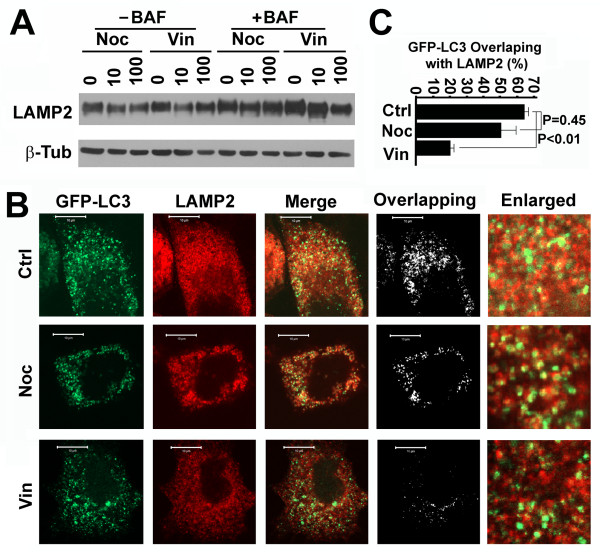
**Vinblastine blocks autophagosome-lysosome fusion on acetylated microtubules**. (**A**) Immunoblotting analysis of LAMP2 levels in HeLa cells treated with different concentrations of nocodazole and vinblastine in the absence or presence of bafilomycin A1 overnight. (**B**) Colocalization analysis of GFP-LC3 punctate foci with lysosomal marker LAMP2 in HeLa cells stably expressing GFP-LC3 treated with nocodazole or vinblastine in the presence of bafilomycin A1 overnight. LAMP2 was visualized with anti-LAMP2 primary antibody and Rhodamine-conjugated secondary antibody. The overlapping analysis was performed with the ImageJ ColocalizeRGB Plugins. (**C**) The fraction of GFP-LC3 overlapping with LAMP2 staining as calculated from images shown in (B) with the ImageJ JACoP Plugins. The data shown is the average and standard deviation of ten randomly selected images in a field of 512 pixels × 512 pixels (23.5 μm × 23.5 μm). The significance of differences was determined by Student's t-test.

To further confirm how vinblastine-induced depolymerization of acetylated microtubules caused the blockade of autophagosomal degradation, we examined the colocalization of GFP-LC3 punctate foci with lysosomes. Neither nocodazole nor vinblastine did not increase the total amounts of lysosomes indicated by LAMP2, a lysosomal membrane-associated protein [30] (Figure 6A). Treatment with bafilomycin A1 caused inhibition of lysosomal activity, but did not change the amount of lysosomal vesicles or LAMP2 levels dramatically (Figure 6A). When lysosomal activity was inhibited, a large number of autolysosomes resulted from fusion of GFP-LC3-labelled autophagosomes with lysosomes were preserved in the control and nocodazole-treated cells causing overlap of more than 50% of GFP-LC3 punctate foci with LAMP2 signal (Figure 6B and 6C). In contrast, vinblastine reduced overlap to less than 20% (Figure 6B and 6C) when the amount of lysosomes were not increase (Figure 6A). This suggested that vinblastine-induced depolymerization of acetylated microtubules impairs the fusion of autophagosomes with lysosomes to form autolysosomes.

## Discussion

To form mature autophagosomes, microtubule-associated LC3I is translocated to sites where it is conjugated with phosphatidylethanolamine to become LC3II that is inserted into isolation membranes [4,5]. The isolation membrane may be pre-assembled in some unidentified subcellular location and transported to sites where substrates and potential cargo exist. Alternatively, small fragments of isolation membrane or some pre-autophagosomal structure may be transported to sites where substrates exist to assemble autophagosomes. Pre-assembled isolation membranes may also remain on site waiting for substrates to appear, or both isolation membrane and substrates may be moved to sites such as microtubule organizing centers to form mature autophagosomes [31]. Independent of the precise mechanism cytoskeletal elements are required for the trafficking of pre-autophagosomal structures, substrates and cargo and mature autophagosomes.

Although both directly bind to the same β-tubulin subunit, paclitaxel prevents while nocodazole promotes depolymerization of normal microtubules. Treatment with either of them results in a similar impact on autophagy. There is no obvious influence on interphase cells cultured under normal conditions, but a similar inhibitory effect on the conversion of LC3I to LC3II in mitotic cells. This suggests that basal levels of autophagy are highly efficient and independent of the status of regular microtubules so that interruption of the dynamics of regular microtubules causes no dramatic impact on overall autophagic influx under steady state conditions. However, consistent with its short duration, but extreme vulnerability to damaged organelles and particularly mitochondria [32], autophagic flux appears to intensify during mitosis [11]. Increased flux makes the conversion of LC3I to LC3II a rate-limiting step so that it is highly responsive to deviations in normal dynamics of microtubules induced by either paclitaxel or nocodazole Inhibition of the conversion of LC3I to LC3II leads to accumulation of LC3I and reduction of total amount of LC3II. This is consistent with the report that autophagosomes can be formed in the absence of intact regular microtubules, but at a significantly lower extent [11].

After autophagosomes mature, they fuse with lysosomes to form autolysosomes. Lysosomes distribute throughout the cytoplasm through anterograde and retrograde movement [33]. Our results show that regular non-acetylated microtubules seem to play no role in the process since their interruption did not cause accumulation of LC3II in the absence of lysosomal inhibitor. This indicates the presence of highly specific cytoskeletal elements are involved in the trafficking of autophagosomes and lysosomes involved in autophagy.

HADC6 is a microtubular deacetylase and regulates microtubule stability [34]. Inhibition of HADC6 enhances microtubular acetylation leading to anterograde trafficking of lysosomes away from centrosomes in addition to an inhibition of autophagosomal biogenesis [18,27,35]. Since microtubular acetylation causes the recruitment of the molecular motors dynein and kinesin-1 to microtubules [18,19], acetylated microtubules may serve for not only the kinesin-dependent anterograde trafficking but also the dynein-dependent retrograde trafficking of either lysosomes or autophagosomes. In addition to the opposite roles in polymerization/depolymerization of regular microtubules by direct binding to β-tubulin, paclitaxel and nocodazole have opposite effects in the acetylation of α-tubulin and stabilization of acetylated microtubules [28]. Paclitaxel enhances, but nocodazole inhibits α-tubulin acetylation and stabilization of acetylated microtubules. However, both of them fail to block autophagosomal degradation. Both paclitaxel and vinblastine enhance the levels of α-tubulin acetylation, but exhibit opposite effects on the polymerization of acetylated microtubules and also opposite roles in autophagosomal degradation. These results suggest that it is not the levels of acetylated α-tubulin that affect autophagosomal degradation. Similar to paclitaxel, nocodazole does not damage the integrity of acetylated microtubules although the total levels of acetylated α-tubulin are reduced [36]. Vinblastine enhances the levels of acetylated α-tubulin, but causes depolymerization of both regular and acetylated microtubules. The treatment not only blocks fusion of LC3II-assoiated autophagosomes with lysosomes, but also reduces efficiency of the LC3I to LC3II conversion similar to paclitaxel or nocodazole. It seems that regular microtubules are involved in, but not essential for the conversion of LC3I to LC3II and degradation of LC3II while acetylated microtubules are required for trafficking of either mature autophagosomes or lysosomes. When autolysosomes were preserved by treatment with bafilomycin A1, a dramatic decrease of number of autolysosomes was observed in cells treated with vinblastine. Our results also confirmed reports [22] that lysosomes may be enriched around centrosomes [27], but the fusion of autophagosomes with lysosomes is not necessarily only around centrosomes.

Based on results from nocodazole treated cells, Fass et al. concluded that microtubules do not support autophagosomal targeting and fusion with lysosomes [11]. As we show here, while nocodazole treatment only causes depolymerization of non-acetylated microtubules, a significant portion of acetylated microtubules are resistant to the treatment. Our current results together with previous reports [9] suggest that cells, particularly in mitosis, have developed a highly-efficient autophagic machinery so that a small fraction of intact acetylated microtubules are sufficient to support fusion and clearance. Thus, nocodazole treatment cannot block the acetylated microtubule-mediated targeting and fusion of autophagosomes with lysosomes. Based on results from cells treated with vinblastine and nocodazole, Kochl et al. [22] emphasized the importance of microtubules, but did not dissect the roles of different subtypes of microtubules in the fusion of autophagosomes with lysosomes. The increase in number of GFP-LC3 punctate foci after vinblastine blockade was interpreted as a stimulation of autophagosomal biogenesis rather than a blockade in clearance [22]. Our results support the idea that the vinblastine-induced increase in number of GFP-LC3 punctate foci is the consequence of autophagosomal accumulation induced by block of autophagosomal fusion with lysosomes and further degradation in lysosomes rather than the stimulation of autophagosomal biogenesis. Since regular microtubules are not essential for autophagosomal biogenesis, the increase of autophagosomal number after vinblastine treatment is more likely caused by continued autophagosomal biogenesis and a blockade of autophagosomal degradation. However, the low degree of overlap of autophagosomes and lysosomes in the presence of high concentrations of nocodazole could be interpreted as the result of nocodazole-induced efficiency of autophagosomal biogenesis rather than a blockade of autophagosome-lysosome fusion as suggested [22]. Although the overnight incubation of microtubule interfering agents in our experiment is longer than the reported two hours [22], the fact that continuous incubation generates no significant more autophagosomes after the 30-minute period of initial incubation suggests that prolonged treatment may cause no significant difference. Since basal levels of autophagy is robust in the entire cell cycle [9], we tested the effects of microtubule-interfering agents on basal autophagy instead of the starvation-induced autophagy as reported [22]. Their result that the effects of microtubule-interfering agents dominate over the starvation-induced effect also suggests whether induced autophagy or basal autophagy is not critical for the investigation of roles of microtubules in autophagy.

## Conclusions

Paclitaxel enhances tubulin acetylation and stabilizes microtubules. Nocodazole depolymerizes non-acetylated microtubules and impairs tubulin acetylation, but does not affect polymerized acetylated microtubules. Vinblastine is able to depolymerize both acetylated and non-acetylated microtubules, but enhances tubulin acetylation. The autophagic responses to the treatments of different microtubular interfering agents reveal that regular non-acetylated microtubules regulate the efficiency of but are not essential for the conversion of LC3I to LC3II. Acetylated microtubules are required for LC3II degradation.

## Methods

### Reagents and antibodies

Microtubule interfering reagents paclitaxel, nocodazole and vinblastine sulfate salt, and lysosomal inhibitor bafilomycin A1 and ammonium chloride (NH_4_Cl) were purchased from Sigma. Monoclonal antibodies against β-actin (c4), acetylated-tubulin (6-11B-1) and LAMP2 (H4B4), polyclonal antibody against β-tubulin (H-235), and FITC- and Rhodamine-conjugated secondary antibodies were purchased from Santa Cruz Biotechnology, Inc. Polyclonal antibodies against LC3 and P62 (SQSTM1) were from Nuvus Bio and ENZO Life Science, respectively.

### Immunoblot analysis

Unless otherwise indicated, lysates were prepared in lysis buffer from cells treated with different drugs overnight, specific times and cell fractions enriched for mitotic or interphase cells as described [32,37]. Immunoblots were prepared from equal amounts of samples separated on SDS-PAGE and analyzed with the indicated antibodies. β-actin served as loading control. Protein band profiles were detected with the Amersham™ ECL™ Plus detection system and a series of images with different exposure times were archived. Data presented in the text for immunoblot or immunohistochemical analysis were representatives of at least three independent experiments. Some bands necessarily appear overexposed because of attempts to display the weakest band. Relative intensities of bands were calculated using ImageJ from scanned images of the respective immunoblot in the linear range and adjusted based on the respective β-actin intensity. The intensity of bands in controls was assigned a unit of 1.

### Immunofluorescence analysis

A stable HeLa cell line expressing GFP-LC3 fusion protein [38] was established as described [9] As described [3], we established a stable HeLa cell line expressing GFP fusion of a mutant version of LC3 (GFP-ΔLC3) that carries a deletion of the 22 amino acid residues of LC3 C-terminus and has the lipid conjugation site Glycine at residue number 120 mutated into Alanine so that it exhibits defective in autophagy initiation. Spread mono-layered interphase cells and round mitotic cells were visualized with a Zeiss LSM510 laser confocal system. GFP-LC3 labeled autophagosomes, MitoTracker Red CMXRos (MitoTracker) labeled mitochondria, primary antibody and corresponding FITC or Rhodamine-conjugated secondary antibody were used to visualize microtubules and lysosomes [32,38]. We specifically demonstrated the relationship between chromosomes and GFP-LC3 previously [9]. Here we used 4',6-diamidino-2-phenylindole (DAPI) instead of TOPRO-3 to label chromosomes and identify different stages of the cell cycle so as to avoid the interference of TOPRO-3 signal to red fluorescent signals. The empty spaces devoid of GFP-LC3 observed in mitotic cells consisted of condensed chromosomes [9]. The chromosomes in paclitaxel-arrested premetaphase cells are closely mingled with GFP-LC3 signals. The staining with MitoTracker generated some diffused and saturated signals in addition to mitochondria, but colocalization of mitochondria with GFP-LC3 punctate foci were shown to be authentic using higher resolution images [9]. The acquired images were exported to Adobe Photoshop, processed and then imported into ImageJ for RGB split and colocalization analysis with a ColocalizeRGB Plugin.

## Abbreviations

ATG: autophagy-related; ATG12: autophagy-related gene 12; ATG5: autophagy-related gene 5; ATG8: autophagy-related gene 8; C19ORF5: chromosome 19 open reading frame 5; GFP-LC3: green fluorescent protein fused light chain 3; HDAC6: histone deacetylase 6; LAMP2: lysosomal-associated membrane protein 2; LC3: light chain 3 of microtubule-associated protein 1A and B, homologue of ATG8; LC3I: soluble form of LC3; LC3II: lipidated form of LC3; MAP1A: microtubule-associated protein 1A; MAP1B: microtubule-associated protein 1B; P62 (SQSTM1): Sequestome I; RASSF1A: RAS association domain family 1A.

## Competing interests

The authors declare that they have no competing interests.

## Authors' contributions

RX and SN executed experiments. WLM participated in design of experiments, interpretation of data and editing the manuscript. LL conceived, designed, and executed experiments, analyzed data, drafted and finalized the edited manuscript. All authors have read and approved the final manuscript.
